# Structure of ice VII with Hirshfeld atom refinement

**DOI:** 10.1107/S2052252525002581

**Published:** 2025-04-25

**Authors:** Roman Gajda, Michał Chodkiewicz, Dongzhou Zhang, Phuong Nguyen, Vitali Prakapenka, Krzysztof Wozniak

**Affiliations:** a Biological and Chemical Research Centre, Department of Chemistry University of Warsaw Żwirki i Wigury 101 Warszawa02-089 Poland; b GeoSoilEnviroCARS University of Chicago 9700 S. Cass Avenue Argonne IL60439 USA; Formby, Liverpool, United Kingdom

**Keywords:** Hirshfeld atom refinement, cubo-ice, ice VII structure, anisotropic displacement parameters

## Abstract

In the refinement of the crystal structures of ice, the best results obtained so far have been with neutron diffraction because the most troublemaking aspects are the hydrogen atoms. In nine out of twenty ice structures, the hydrogen atoms are disordered, which makes proper refinement more difficult. In our previous paper describing the structure of ice VI we proved that, using Hirshfeld atom refinement (HAR) based on synchrotron X-ray data, it is possible to obtain results comparable with those from neutron experiments. In this work, we investigate another structure of high-pressure disordered ice, cubo-ice (ice VII). Single crystals of cubo-ice were grown under pressure in diamond anvil cells. X-ray diffraction measurements were conducted at a synchrotron source facility (APS, University of Chicago, USA) as well as on our regular in-house laboratory diffractometer with Ag radiation. The data collected were further refined with HAR. Comparison of the structural parameters obtained with those derived from neutron diffraction showed very good agreement in terms of bond lengths and fairly good agreement in terms of hydrogen atom ADPs. We were also able to perform unconstrained refinements with various split-atom models.

## Abstract 2

The crystal structure of cubo-ice (ice VII) has been established by single-crystal X-ray diffraction using both synchrotron and laboratory data collected at high pressure. X-ray diffraction data in both cases were refined with Hirshfeld atom refinement. Various structural models including those with ‘split’ positions of atoms were refined.

## 1. Introduction

Water ice exhibits a very complex phase diagram (Petrenko & Whitworth, 2002 ▸; Bartels-Rausch *et al.*, 2012 ▸) which is still under dynamic development (*e.g.* Hansen, 2021 ▸) and many questions on the structures of water ice still remain open (Loerting *et al.*, 2020 ▸). Ice VII (cubo-ice) is one of at least twenty currently known crystalline forms of ice. It is hypothesized to be present in large icy satellites (*e.g.* Jupiter’s moon Europa) and some exoplanets but was also found as inclusions in diamonds in Earth’s mantle (Tschauner *et al.*, 2018 ▸). It belongs, with nine other structures of ice, to ices with disordered hydrogen atoms. The structure of ice VII consists of two interpenetrating (but not interconnecting) hydrogen-bonded networks (Kamb & Davis, 1964 ▸), each of them resembles the one known from the ordinary ice structure. A basic model of the structure assumes that the oxygen atoms are located at highly symmetric special positions (43*m*) which leads to too-short O—D distances: about 0.89 Å (Kuhs *et al.*, 1984 ▸); subsequent measurements by Jorgensen & Worlton (1985 ▸) yielded a larger [0.943 (2) Å] but still too-small value. This observation initialized a search for a more plausible model, leading to multiple analyses of the problem over the last 40 years. Neutron diffraction is a dominant technique for determining the structures of ice (Komatsu, 2022 ▸). Although in practice all the experimental studies on the details of the ice VII structure were performed with the help of neutron diffraction, in this work we test the possibility of extracting structural information for this kind of system using single-crystal X-ray diffraction (XRD). The development of more advanced models of atomic form factors based on aspherical atomic densities greatly improved the accuracy of hydrogen atom parameters determined from XRD experiments (Woińska *et al.*, 2016 ▸; Jha *et al.*, 2020 ▸). Hirshfeld atom refinement (HAR) (Capelli *et al.*, 2014 ▸; Jayatilaka & Dittrich, 2008 ▸; Chodkiewicz *et al.*, 2020 ▸) is, in principle, the most accurate of them. It utilizes an electron density calculated quantum-mechanically for the system of interest and the electron density is then split into atomic contributions with Hirshfeld partition (Hirshfeld, 1977 ▸); application of other partitions is also possible (Chodkiewicz *et al.*, 2020 ▸; Chodkiewicz & Woźniak, 2025 ▸). We have successfully applied HAR previously to study the crystal structure of ice VI (Chodkiewicz, Gajda *et al.*, 2022 ▸).

## 2. Structure of cubo-ice (ice VII)

**Figure 1:**
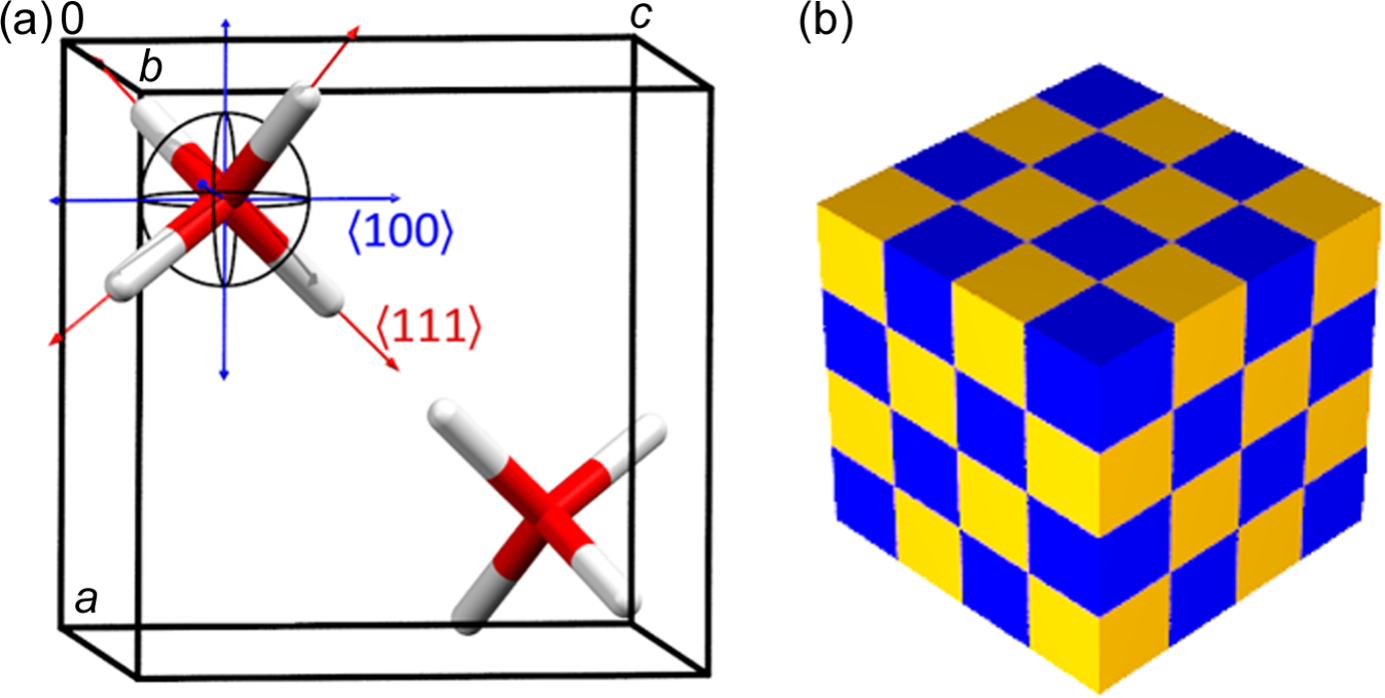
(*a*) Ice VII unit-cell content, single-site model and the 〈100〉 and 〈111〉 families of oxygen displacement directions. (*b*) The boxes represent unit cells where the same-colour unit cells contain water molecules belonging to the same hydrogen-bonding framework.

Cubo-ice crystallizes in the cubic space group *Pn*3*m*. The unit cell (lattice constant *ca* 3.36 Å) contains only two disordered water molecules [Fig. 1 ▸(*a*)]. They belong to the same (one of two) hydrogen-bonded framework. Such a framework corresponds to the content of a group of unit cells connected by sharing only one edge [forming a 3D chessboard-like structure, Fig. 1 ▸(*b*)]. The asymmetric unit contains only one oxygen atom occupying the (simplified model) 2*a* Wyckoff position (1/4, 1/4, 1/4) with the point-group symmetry 43*m*, and one hydrogen atom at the 8*e* site (3*m* symmetry) at (*x*, *x*, *x*), *x* ≃ 0.41, with an occupancy of 1/2.

A structure determination by Kuhs *et al.* (1984 ▸) revealed that the model described above is too simplistic and leads to unphysical geometry of water molecules. A split of the oxygen atom position was suggested and included in refinement with the anharmonic model of its atomic displacement. The refinement suggested shift of the oxygen atom from the 2*a* special position along the 〈100〉 family of (6) directions (see Fig. 1 ▸). Subsequent partially constrained refinements suggested an oxygen shift by about 0.1 Å.

Subsequent work by Jorgensen & Worlton (1985 ▸) led to the suggestion that the deuterium atom position is also split; the original *x*, *x*, *x* position was replaced with *x*, *x*, *z*, which gives two independent situations for *z* > *x* and *z* < *x*. The model for *x* > *z* with isotropic displacement for deuterium gave a lower *R* value than the one with *x* < *z * and also lower than an anisotropic model without such displacement. Displacement of the oxygen atoms along 〈100〉 up to 0.1 Å did not change the agreement factor, but refinement of the displacement was not possible.

Nelmes *et al.* (1998 ▸) studied a multisite displacement model for ice VII. They also measured data for ice VIII, which undergoes a phase transition to ice VII on heating. The ice VIII structure is similar to the ice VII structure, the main difference being the ordering of hydrogen atoms in the case of ice VIII. Due to a hysteresis in critical temperature (*T*_c_) (Pruzan *et al.*, 1993 ▸), it is possible to measure data for both phases at the same temperature range under the same pressure. The anisotropic displacement parameters (ADPs) obtained with the ice VIII structure refinement were then used in the ice VII refinement. It was possible to examine several displacement directions for hydrogen and oxygen. Regardless of the oxygen displacement direction, its magnitude was 0.135 (10) Å. It was found that the only displacement direction for oxygen atoms that leads to a reasonable geometry for a water molecule is along 〈111〉. The result is quite different from previous reports.

The two hydrogen-bonded frameworks in ice VIII are slightly displaced relative to each other along the *c* direction. Such displacement in ice VII could be locally preserved if oxygen atoms are shifted along 〈100〉. A shift along 〈111〉 would introduce quite different hydrogen bonding to 〈100〉, the former would lead to two sets of hydrogen bond oxygen–oxygen distances that are shorter and longer than those in ice VIII by about 0.1 Å. A shift in 〈100〉 would preserve a unimodal distribution of the distances.

Subsequent computational studies by Kuo & Klein (2004 ▸) and Knight & Singer (2009 ▸) used periodic DFT calculations for multiple possible configurations of ice structures. Both studies did not note the bimodal distribution of hydrogen bond O⋯O distances which would support the model with a shift along 〈111〉. The complexity of the structure composed of a combination of such local configurations was stressed. Knight & Singer (2009 ▸) also studied oxygen atom displacement and found that it is displaced in the 〈100〉 direction with a maximum of the density distribution near 0.071 Å from the (1/4, 1/4, 1/4) position.

In the work by Komatsu *et al.* (2015 ▸), the ice VII structure was refined with an unsplit oxygen atom and deuterium hydrogen atom split into three positions with the coordinates of one of the atoms given by *x*, *x*, *z* with *z* < *x*: the same model which gave the best agreement factors in the study by Jorgensen & Worlton (1985 ▸).

Recent experimental work by Yamashita *et al.* (2022 ▸) based on powder and single-crystal neutron diffraction revealed quite a different picture. A three-dimensional atomic distribution was reconstructed using the maximum entropy method. It was observed that the distribution of deuterium atoms has a ring-like structure around the 〈111〉 directions and that the oxygen distribution is extended towards O—D bonds, which corresponds to displacement of the oxygen atom along the 〈111〉 direction.

Despite numerous studies, details of the ice VII structure remained (or maybe still remain) a puzzle. In this work, we attempt to analyse the ice VII structure with XRD.

## 3. Results

In our analysis, we first analyse the ‘single-site’ model of the cubo-ice structure, which means the ordered oxygen atom and the hydrogen atom at the 3*m* site symmetry. Subsequently, more complex models are considered.

**Figure 2:**
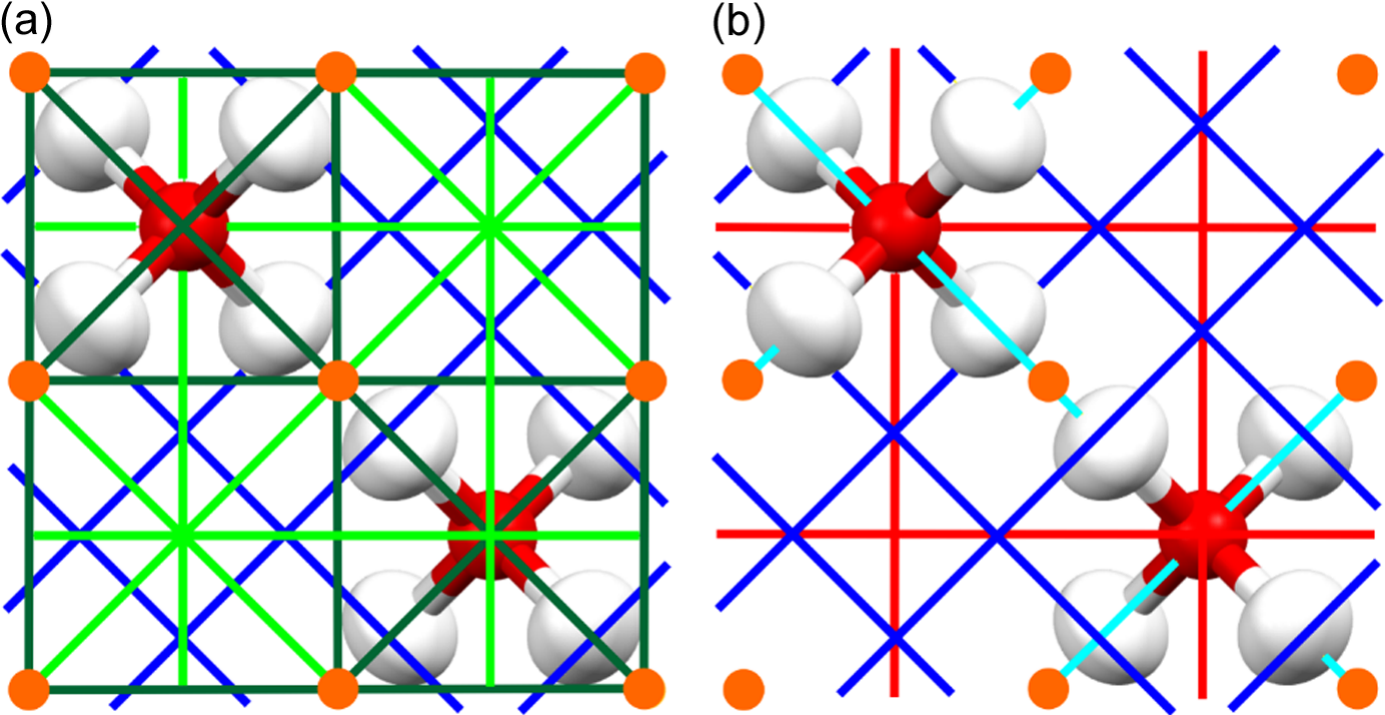
Selected symmetry elements in the cubo-ice unit cell. Glides and mirror planes have been omitted for clarity. (*a*) Combination of two- and threefold axes. (*b*) Combination of three- and fourfold axes. Orange circles – inversion centres, light green lines – twofold axes, dark green lines – twofold inversion axes, light blue lines – threefold axes, dark blue lines – threefold inversion axes, red lines – fourfold inversion axes.

The unit cell of the single-site model of cubo-ice is presented in Fig. 2. Because it is hard to clearly depict all the existing elements of symmetry in a clear way at once, this figure is divided into Fig. 2 ▸(*a*) and Fig. 2 ▸(*b*), which should be considered together.

There are four structures described as ice VII (three D_2_O and one H_2_O) currently deposited in the ICSD (Kamb & Davis, 1964 ▸; Kuhs *et al.*, 1984 ▸; Jorgensen & Worlton, 1985 ▸; Komatsu *et al.*, 2015 ▸). All of them were determined based on powder data only. Only Jorgensen & Worlton (1985 ▸) present ADPs for the hydrogen atom.

To collect data for this paper, we conducted a series of measurements for single crystals of cubo-ice that consisted either D_2_O, H_2_O, or a 50%/50% mix of D_2_O and H_2_O ice VII. Data were mainly collected at the APS synchrotron radiation facility in Argonne and one dataset came from our laboratory in Warsaw.

The literature data which work for us as a benchmark (Jorgensen & Worlton, 1985 ▸) are powder data, whereas our data came from single-crystal measurements. The neutron structure of ice VII was determined based on 35 Bragg reflections. Our single-crystal measurements for which we were unable to determine the ADPs [APS-D_2_O and APS-H_2_O (a)] have fewer, just 24 and 22 independent reflections, respectively. The other datasets were more complete. The number of independent reflections varied from 33 to 45, which allowed us to obtain the ADPs. For those datasets, refinements with the oxygen and hydrogen positions split were also performed.

### 3.1. Single-site model refinements

Measurement descriptions and O—H bond lengths for the single-site refinements and reference neutron structure are presented in Table 1 ▸. ADPs and similarity indicators comparing X-ray- and neutron-derived hydrogen atom ADPs are given in Table 2 ▸.

The two datasets for which it was impossible to refine hydrogen atom ADPs have large standard deviations (0.2–0.3 Å) of O—H bond lengths and contain a smaller number of reflections than other sets (Table 1 ▸). They are not analysed further. Only one more parameter is necessary to switch from an isotropic to an anisotropic description of hydrogen atom ADPs. X-ray measurements were performed at a lower pressure than the neutron one and for crystals of varying D/H composition, therefore high similarity between X-ray and the reference measurement is not expected.

**Figure 3:**
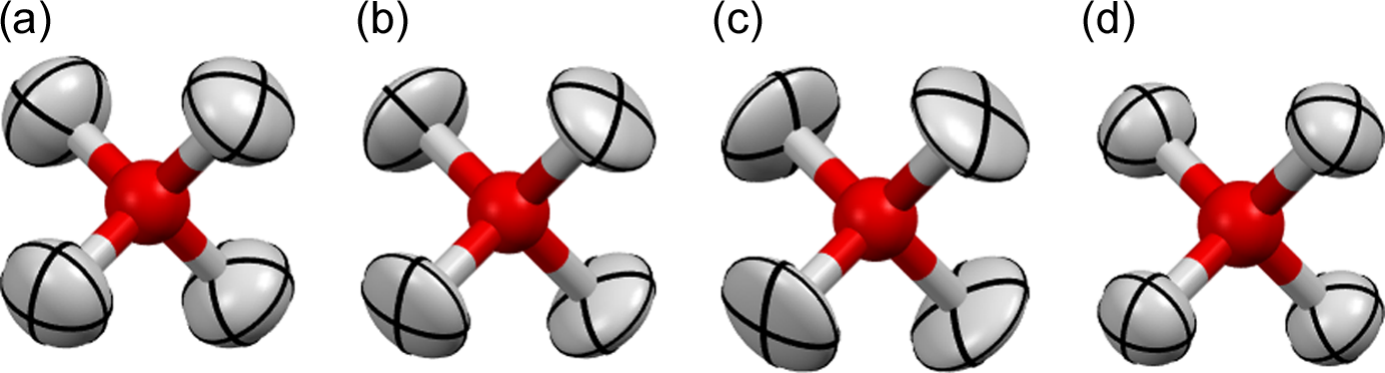
Disordered H_2_O molecules in ice VII, (*a*)–(*c*) from HAR: (*a*) APS-H_2_O (b), (*b*) APS-mix (a), (*c*) Home-D_2_O and (*d*) Neutron data D_2_O.

While the X-ray-derived O—H bond length is similar [0.95 (2) Å for D_2_O] to the one from neutron diffraction [0.943 (2)], the ADPs are not so similar (Fig. 3 ▸ and Table 2 ▸), especially in the case of the D_2_O X-ray measurement. The comparison of atomic displacement tensor components perpendicular/parallel to the O—H bond (*U*_⊥_/*U*_∥_ in Table 2 ▸) indicates that the X-ray-derived ADPs are larger in the direction perpendicular to the O—H bond. In the cases of D_2_O and H_2_O/D_2_O mix, they are also quite small in the bond direction. The X-ray-derived hydrogen atom ADPs were compared with those derived from neutron diffraction using the *S*_12_ similarity index (Whitten & Spackman, 2006 ▸) and the η_r_ rescaled overlapping coefficient (Chodkiewicz *et al.*, 2024 ▸) which can be seen as a percentage difference between the probability distributions for atomic displacements. The difference is between 12 and 25% and it is hard to tell what the source of the discrepancy is.

### 3.2. Multisite refinements

**Figure 4:**
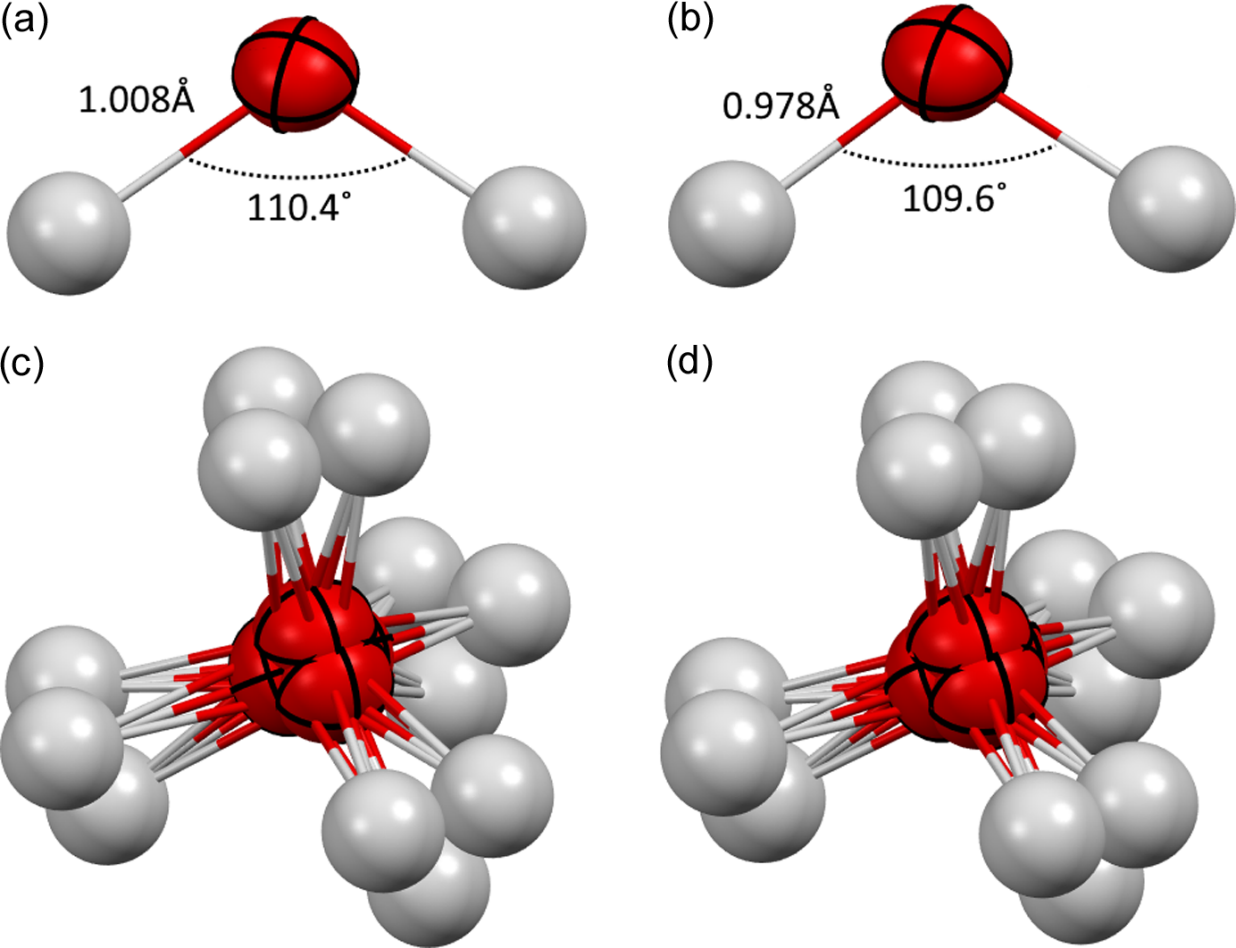
Structures refined with the oxygen atom split in the 〈111〉 family of directions. (*a*, *b*) Geometries of selected configurations of the water molecule. (*c*, *d*) Disordered water molecule. Refinements for (*a*) and (*c*) H_2_O; (*b*) and (*d*) D_2_O.

Earlier publications on ice VII reported several ways of performing split atom refinements. We have applied oxygen atom splitting in the 〈100〉 and 〈111〉 directions and two models of the hydrogen atom split, from *x*, *x*, *x* to *x*, *x*, *z* with *x* < *z* or with *x* > *z*. The model with hydrogen atom split and isotropic hydrogen atom displacement parameters uses the same number of parameters as the model with ADPs and no split. Combinations of five oxygen models with four hydrogen models were tested and examined. For oxygen: split in the 〈100〉 direction with (1) isotropic, (2) anisotropic ADPs, (3) no split; split in the 〈111〉 direction with (4) isotropic, (5) anisotropic ADPs. For hydrogen: (1) anisotropic ADPs, no split position; isotropic ADPs with split position *x*, *x*, *z* with (2) *x* < *z* and (3) with *x* > *z* and (4) no split position. About half of the combinations were not preserved during refinement (*i.e.* switched to some other model), see Table 3 ▸. Our goal was to check for some repeating patterns in the refinements across various datasets, but none were discovered. For example, it is not possible to refine H_2_O with the 〈100〉 direction oxygen split, but it is possible to do it for D_2_O and the D_2_O/H_2_O mix; it is also not possible to refine any structure with the 〈111〉 direction oxygen split when the isotropic model of ADPs is used, but it is possible with the anisotropic one. In many cases, the possibility of performing a refinement with a certain model for oxygen depends on the model for hydrogen and vice versa. In principle, meaningful analysis of the result of the split model would require great caution especially when the differences in figures of merits are relatively small – taking into account the reliability of the standard deviations of the measured intensities, the exact way numerical algorithms of the refinement program optimization procedure work and the rules the program uses for shifting atoms into special positions. Reported *wR*_2_ agreement factors (Table 4 ▸) are not always comparable with each other because the *SHELXL* weighting scheme was used with parameters that vary from refinement to refinement. However some can be compared and, for example, *wR*_2_ for two variants of hydrogen atom split (*z* < *x* and *z* > *x*) for the H_2_O/D_2_O mix is slightly lower (8.24 versus 8.28) when *z* > *x* and the oxygen atom is modelled with 〈100〉 direction split, but the opposite situation takes place when the oxygen atom is modelled without such a split. Also for D_2_O the lower *wR*_2_ is for the *z* < *x* model. Since the differences in *wR*_2_ are rather small and not consistent (they differ from model to model), *wR*_2_ does not seem to clearly indicate any preferred model. Both computational and experimental studies suggest that the local structure of ice VII is too complicated to be described with the simple split models. Therefore we did not try to statistically analyse which of the models tested is closer to reality. In the case of the 〈111〉 shift, the refinement ended with the equivalent 〈111〉 shift in some cases (for mixed ice data). For H_2_O it was possible to refine oxygen with an anisotropic atomic displacement model with both models of hydrogen split. It was also possible in the case of D_2_O, but refinement led to shifting the oxygen atom into the original special position. The resulting model allowed us to choose water molecule configurations with a geometry (Fig. 4 ▸) that is relatively close to the geometry of water in ice VIII (Kuhs *et al.*, 1984 ▸) in terms of O—H bond length (0.978 versus 0.969 Å for ice VIII) and H—O—H angle (109.6 versus 105.6° for ice VIII). This does not mean that the model is the preferred one, as computational studies have shown that a combination of water configurations can lead to different average oxygen atom displacements yet preserve the typical structure of the water molecules involved (Knight & Singer, 2009 ▸).

### 3.3. Concluding remarks

Similar to our previous study on ice VI (Chodkiewicz, Gajda *et al.*, 2022 ▸), accurate information on the high-pressure disordered ice structure (ice VII this time) was extracted from XRD data using HAR. Limitations introduced by high-pressure measurement and disorder in the structure of the system make such studies challenging. Though not all of the collected datasets allowed for obtaining high-accuracy structures, for some of them the agreement with neutron ones in terms of bond lengths was very good (*e.g.* 0.95 Å and 0.93 Å versus 0.943 Å) and it was possible to obtain anisotropic ADPs of the hydrogen atom which qualitatively agree with those from neutron measurements. We were also able to perform refinements with a split-atom model for oxygen and hydrogen and anisotropically refine ADPs of the oxygen in the split-site model. In practice, the disorder in ice VII is probably too complicated to be explained with the simple ‘split’ models we used in the refinements. This work indicates that XRD might become a valuable source of structural information on ice structures despite neutron diffraction domination in the field. For example, the X-ray refinements for ice VII, similar to the neutron ones, showed that a single-site model gives too-short O—H bonds, indicating that a more complicated model is needed.

## 4. Experimental

### 4.1. Crystallization of the sample

Each single crystal of ice VII measured in this research was prepared separately in a Merrill–Bassett type diamond anvil cell (DAC). This means in particular that the DAC placed on the laboratory diffractometer or in the synchrotron beamline contained only one piece of single crystal. The pressure cell was filled with only pure liquid: D_2_O, H_2_O, or a 50:50 mix of D_2_O and H_2_O. No pressure medium was used. The pressure in the DAC was increased or decreased manually by three screws. Each single crystal was grown *in situ* in the pressure chamber in a few steps. At the first step, the pressure was increased to achieved a pressure point where the liquid sample crystallizes into ice VI. Then, a stream of very hot air was applied to the DAC and the pressure was further raised. The temperature inside the pressure chamber was estimated by a thermocouple attached to the DAC. Its tip was touching the stainless steel gasket in which the sample was squeezed between two diamond anvils. The temperature inside the pressure cell, determined in this way, was only approximate but its increments allowed us to find out where on the phase diagram the sample is at that time. Simultaneously, the pressure in the DAC was gradually increased. The goal was to achieve as high pressure as possible but the polycrystalline form should still be meltable at a temperature of the hot stream below 400°C. The processes of melting the polycrystalline form were observed constantly under microscope. To protect the fragile parts of the microscope optics, a hot stream with a temperature higher than 400°C was impossible to achieve. When the polycrystalline form, with exception of one grain, was melted, the whole pressure chamber was slowly cooled down. As a result the separate single crystal grain was growing and filling in the whole space. The process of cooling down from the temperature well above 300°C to room temperature was slow and took hours. Due to the fact that the DAC was made of steel, thermal expansion of the material had a strong influence on pressure inside the pressure chamber. When the DAC was within the hot stream under the microscope it was not possible to determine the pressure exactly. However, when the DAC was cooled down to *ca* room temperature, the pressure inside pressure chamber dropped significantly. That is why, even if the pressure seemed to be significant during the melting of the polycrystalline form at high temperature, after cooling it was only close to *ca* 2 GPa.

### 4.2. Pressure determination in the DAC

The pressure of the sample inside the DAC was determined by the ruby fluorescence method, collected through an optical-fibre coupled HRS-300 spectrometer (Teledyne) with a 1200 g mm^−1^ grating (Zhang *et al.*, 2022 ▸). The ruby pressure scale from Dewaele *et al.* (2008 ▸) was used.<!?tpb=-4pt>

### 4.3. Synchrotron X-ray diffraction measurements

Single-crystal XRD data collection at high pressure was carried out at the experimental station 13-BM-C at the Advanced Photon Source, Argonne National Laboratory (λ = 0.434 Å). Measurements were conducted at room temperature and under non-ambient pressures. Single crystals were grown in DACs (Merrill–Bassett type). The diffraction images were merged and reduced to *hkl* files using the *APEX3* software package (Bruker). The pressure value of particular experiments varied between 1.8 and 2.1 GPa, determined using the ruby fluorescence method.<!?tpb=-4pt>

### 4.4. In-house measurement

The in-house data collection was conducted at ambient temperature and with the use of a DAC (Merrill–Bassett type). Measurements were conducted using a diffractometer equipped with an Ag X-ray microfocus source (*K*α = 0.5609 Å). Data reduction was performed using the *Crys­AlisPro* software (Rigaku Oxford Diffraction, 2014 ▸). The structure was solved and refined with *SHELXS* (Sheldrick, 2008 ▸) and *SHELXL* (Sheldrick, 2015 ▸), respectively, within the *Olex2* suite (Dolomanov *et al.*, 2009 ▸). The pressure at which the measurement was conducted was determined to be 2.2 GPa.<!?tpb=-4pt>

### 4.5. Data analysis with use of HAR

For HAR, a locally modified version of *Olex2* (Dolomanov *et al.*, 2009 ▸) was used in the refinements incorporating tools based on a development version of the *DiSCaMB* library (Chodkiewicz *et al.*, 2018 ▸) which generates files with atomic form factors in .tsc format (Kleemiss *et al.*, 2021 ▸; Midgley *et al.*, 2019 ▸). Such files are then imported into *Olex2* and used in the refinement. Details of the implementation are given by Chodkiewicz, Pawledzio *et al.* (2022 ▸). A density functional method with a B3LYP functional and *cc-pVTZ* basis set was used for calculation of the electron densities. Quantum mechanical calculations were performed with *ORCA* (Neese *et al.*, 2020 ▸). The crystal-field effects were represented in quantum mechanical calculations by surrounding the water molecule with its four nearest-neighbour water molecules. While there are multiple ways such a cluster can be constructed, we have chosen a cluster with the arrangement corresponding to the structure of ice VIII. For refinements with the split-atom model, the atomic form factors calculated for single-site model were used.

## Supplementary Material

CIFs for all refinements. DOI: 10.1107/S2052252525002581/lt5074sup1.zip

Supporting tables. DOI: 10.1107/S2052252525002581/lt5074sup2.pdf

CCDC references: 2413736, 2413737, 2413738, 2413739, 2413740, 2413741, 2413743, 2413744, 2413745, 2413746

## Figures and Tables

**Table 1:** Measurement descriptions and comparison of O—H bond lengths for structures refined using HAR

Type of measurement	Pressure (GPa)	Unit-cell parameter (Å)	O—H bond length (Å)	Independent reflections
APS-D_2_O	2.3	3.3661 (4)	0.9 (3)	24
APS-H_2_O (a)	2.1	3.3891 (6)	0.9 (2)	22
APS-H_2_O (b)	2.1	3.3887 (5)	0.91 (3)	33
APS-mix (a)	1.8	3.3891 (3)	0.93 (4)	45
Home-D_2_O†	2.2	3.3769 (4)	0.95 (2)	38
Neutron-D_2_O‡	2.6	3.3501 (1)	0.943 (2)	35

† Data collected on our laboratory diffractometer with an Ag X-ray source.

‡ Jorgensen & Worlton (1985 ▸).

**Table 2:** Comparison of hydrogen atom ADPs for structures refined using HAR and reference values *U*_⊥_/*U*_∥_ – perpendicular/parallel components of *U* tensor of the O—H bond; *S*_12_ and η_r_ – ADPs similarity indices.

	ADPs of hydrogen atoms				
	*U*_11_/*U*_12_	*U*_⊥_/*U*_∥_	*U*_iso_	*S*_12_	η_r_
APS-D_2_O	isotropic		0.04 (8)	–	
APS-H_2_O (a)	isotropic		0.03 (5)	–	
APS-H_2_O (b)	0.043 (5)/−0.006 (7)	0.050 (6)/0.028 (6)	0.043 (5)	1.35	12.3
APS-mix (a)	0.041 (7)/−0.012 (12)	0.052 (11)/0.015 (10)	0.040 (8)	3.04	16.8
Home-D_2_O†	0.047 (5)/−0.018 (6)	0.065 (6)/0.012 (6)	0.047 (5)	6.59	25.1
Neutron-D_2_O‡	0.03255/−0.00431	0.03686/0.02393	0.02824		

† Data collected on our laboratory diffractometer with an Ag X-ray source.

‡ Jorgensen & Worlton (1985 ▸).

**Table 3:** Possibility to perform refinement for various structural models Refinement resulting in (×) changing the structural model and (•) preserving the model. The following colour coding was used: black – D_2_O/H_2_O mix, red – D_2_O and blue – H_2_O.

		H iso		
	H aniso	*z* < *x*	*z* > *x*	Iso
O 〈100〉 iso	•××	••×	••×	••×
O 〈100〉 iso	•××	×××	×××	••×
O iso	•••	•••	•••	•••
O 〈111〉 iso	×××	×××	×××	×××
O 〈111〉 aniso	•×•	××•	×••	••×

**Table 4:** *wR*_2_ agreement factor for various structures and structural models (see text) In the case of the H_2_O/D_2_O mix, only the bold numbers are comparable (due to similar weighting scheme parameters); for D_2_O all models are comparable; and for H_2_O the numbers in bold are comparable and, separately, the numbers in italics.

	H_2_O/D_2_O mix				D_2_O				H_2_O			
	H aniso	H iso, *z* < *x*	H iso, *z* > *x*	H iso, *z* = *x*	H aniso	H iso, *z* < *x*	H iso, *z* > *x*	H iso, *z* = *x*	H aniso	H iso, *z* < *x*	H iso, *z* > *x*	H iso, *z* = *x*
O 〈100〉 iso	8.26	**8.28**	**8.24**	**8.29**	–	4.05	4.22	4.29	–	–	–	–
O 〈100〉 aniso	8.23	–	–	7.15	–	–	–	4.19	–	–	–	–
No split	9.16	**9.18**	**9.23**	**9.36**	4.21	4.25	4.49	4.60	**12.15**	**12.17**	12.03	13.81
O 〈111〉 iso	–	–	–	–	–	–	–	–	–	–	–	–
O 〈111〉 aniso	7.57	–	–	7.75	–	–	4.24	4.28	*12.0*	*11.94*	12.06	–
