# Supplementary material for: Structure of ice VII with Hirshfeld atom refinement
Source: IUCrJ. 2025 Apr 25;12(Pt 3):288–94. doi: 10.1107/S2052252525002581 (PMC12044852; doi:10.1107/S2052252525002581)
Supplement: Supplementary file 2 [file m-12-00288-sup2.pdf]

IUCrJ

Volume 12 (2025)

Supporting information for article:

Structure of ICE VII with Hirshfeld atom refinement

Roman Gajda, Michał Chodkiewicz, Dongzhou Zhang, Phuong Nguyen, Vitali Prakapenka and Krzysztof Wozniak

Table S1 Crystal structure data for ice VII.

Data set	APS-D_2O	APS-H_2O(a)	APS-H_2O(b)
Pressure (GPa)	2.3	2.1	2.1
A (Å	3.3661 (4)	3.3891 (6)	3.3887 (5)
V (Å^3)	38.14 (1)	38.93 (1)	38.91 (1)
F(000)	19.997	19.997	19.997
D_x (Mg m^{-3})	1.569	1.537	1.538
Radiation type	λ = 0.434 Å	λ = 0.434 Å	λ = 0.434 Å
μ (mm^{-1})	0.06	0.06	0.06
Measured, independent, observed [$I \geq 2u(I)$] refl.	72, 24, 16	83, 22, 16	246, 33, 24
R_{int}	0.034	0.023	0.050
θ values (°)	θ_{max} = 27.7, θ_{min} = 5.2	θ_{max} = 23.3, θ_{min} = 7.4	θ_{max} = 26.9, θ_{min} = 5.2
$(\sin \theta/\lambda)_{max}$ (Å^{-1})	1.071	0.910	1.043
Range of h, k, l	$h = -5\rightarrow3$, $k = -4\rightarrow5$, $l = -2\rightarrow7$	$h = -4\rightarrow3$, $k = -5\rightarrow3$, $l = -4\rightarrow4$	$h = -4\rightarrow5$, $k = -5\rightarrow4$, $l = -4\rightarrow3$
Refinement on	F^2	F^2	F^2
$R[F^2 > 2\sigma(F^2)]$, $wR(F^2)$, S	0.060, 0.207, 1.00	0.045, 0.293, 2.75	0.044, 0.135, 1.15
No. of reflections	24	22	33
No. of parameters	4	4	5
Weighting scheme	$w = 1/[\sigma^2(F_o^2) + (0.082P^*)^2 + 0.1865P^*]$	$w = 1/[\sigma^2(F_o^2) + (0.0648P^*)^2 + 0.012P^*]$	$w = 1/[\sigma^2(F_o^2) + (0.0942P)^2]$
$(\Delta/\sigma)_{max}$	0.0002	0.0001	0.001
Δ_{max}, Δ_{min} (e Å^{-3})	0.52, -1.16	0.35, -0.34	0.16, -0.31

*P = $P = (F_o^2 + 2F_c^2)/3$

Table S2 Crystal structure data for ice VII - continuation

Data set	APS–mix (a)	APS–mix (b)	Home-D_2O
Pressure (GPa)	1.8	1.8	2.2
A (Å	3.3891 (3)	3.3891 (3)	3.3769 (4)
V (Å^3)	38.93 (1)	38.93 (1)	38.51 (1)
F(000)	19.997	19.997	20.010
D_x (Mg m^{-3})	1.537	1.537	1.554
Radiation type	λ = 0.434 Å	λ = 0.434 Å	λ = 0.5609 Å
μ (mm^{-1})	0.06	0.06	0.10
No. of measured, independent and observed [$I \geq 2u(I)$] reflections	204, 45, 28	204, 45, 28	735, 38, 27
R_{int}	0.039	0.039	0.064
θ values (°)	θ_{max} = 31.3, θ_{min} = 6.4	θ_{max} = 31.3, θ_{min} = 6.4	θ_{max} = 36.0, θ_{min} = 8.3
$(\sin \theta/\lambda)_{max}$ (Å^{-1})	1.198	1.198	1.047
Range of h, k, l	$h = -4\rightarrow6$, $k = -4\rightarrow8$, $l = -2\rightarrow2$	$h = -4\rightarrow6$, $k = -4\rightarrow8$, $l = -2\rightarrow2$	$h = -4\rightarrow4$, $k = -7\rightarrow6$, $l = -5\rightarrow5$
Refinement on	F^2	F^2	F^2
$R[F^2 > 2\sigma(F^2)]$, $wR(F^2)$, S	0.025, 0.092, 2.17	0.023, 0.087, 2.08	0.017, 0.043, 1.43
No. of reflections	45	45	38
No. of parameters	5	6	5
Weighting scheme	$w = 1/[\sigma^2(F_o^2) + (0.010P^*)^2 + 0.0001P^*]$	$w = 1/[\sigma^2(F_o^2) + (0.010P^*)^2 + 0.0001P^*]$	$w = 1/[\sigma^2(F_o^2) + (0.010P^*)^2 + 0.0002P^*]$
$(\Delta/\sigma)_{max}$	0.0003	0.0001	0.0001
Δ_{max}, Δ_{min} (e Å^{-3})	0.18, -0.16	0.21, -0.15	0.08, -0.14

*P = $P = (F_o^2 + 2F_c^2)/3$

Table S3 Atomic positions in structures of cubo-ice (ice VII).

Measurement	Atom	x	y	z
**APS–D_2O 2.3GPa**	O	0.25	0.25	0.25
	H	0.41(5)	0.41(5)	0.41(5)
**APS–H_2O (a) 2.1GPa**	O	0.25	0.25	0.25
	H	0.41(3)	0.41(3)	0.41(3)
**APS–H_2O (b) 2.1GPa**	O	0.25	0.25	0.25
	H	0.400(5)	0.400(5)	0.400(5)
**APS–mix (a) 1.8GPa**	O	0.25	0.25	0.25
	H	0.409(7)	0.409(7)	0.409(7)
**APS-mix (b) 1.8GPa**	O	0.204(4)	0.25	0.25
	H	0.406(8)	0.406(8)	0.406(8)
***Home–D_2O 2.2GPa**	O	0.25	0.25	0.25
	H	0.409(3)	0.409(3)	0.409(3)
****Neutron data D_2O**	O	0.25	0.25	0.25
	H	0.412515	0.412515	0.412515
