# Supplementary material for: Structure of ice VII with Hirshfeld atom refinement
Source: IUCrJ. 2025 Apr 25;12(Pt 3):288–94. doi: 10.1107/S2052252525002581 (PMC12044852; doi:10.1107/S2052252525002581)

checkCIF/PLATON report

Structure factors have been supplied for datablock(s) solve2

THIS REPORT IS FOR GUIDANCE ONLY. IF USED AS PART OF A REVIEW PROCEDURE FOR PUBLICATION, IT SHOULD NOT REPLACE THE EXPERTISE OF AN EXPERIENCED CRYSTALLOGRAPHIC REFEREE.

No syntax errors found. CIF dictionary Interpreting this report

Datablock: solve2

Bond precision: = 0.0000 A Wavelength=0.56087

Cell:	a=3.3769(4)	b=3.3769(4)	c=3.3769(4)
	alpha=90	beta=90	gamma=90
Temperature:	286 K		

	Calculated	Reported
Volume	38.508(14)	38.508(8)
Space group	P n -3 m	P n -3 m
Hall group	-P 4bc 2bc	-P 4bc 2bc
Moiety formula	H2 O	H2 O1
Sum formula	H2 O	H2 O
Mr	18.02	18.01
Dx,g cm-3	1.554	1.554
Z	2	2
Mu (mm-1)	0.101	0.102
F000	20.0	20.0
F000’	20.01	
h,k,lmax	7,7,7	3,6,4
Nref	48	38
Tmin,Tmax		
Tmin’		

Correction method= Not given

Data completeness= 0.792 Theta(max)= 35.960

R(reflections)= 0.0179(27) wR2(reflections)= 0.0421(38)

S = 1.419 Npar= 5

The following ALERTS were generated. Each ALERT has the format
test-name_ALERT_alert-type_alert-level.
Click on the hyperlinks for more details of the test.

Alert level A

```
PLAT029_ALERT_3_A _diffrn_measured_fraction_theta_full value Low .      0.909 Why?
```

Author Response: It is caused by the fact that sample was closed in Diamond Anvil Cell and as a result access to the reciprocal psace was restricted.

Alert level B

```
PLAT088_ALERT_3_B Poor Data / Parameter Ratio ....................       7.60 Note
```

Author Response: low number of reflections has been measured, pourpose of the refinement was to check results of the refinement for the model used inspite of the poor Data / Parameter Ratio

```
PLAT113_ALERT_2_B ADDSYM Suggests Possible Pseudo/New  Space Group      Im-3m Check
              Check Model Parameter Symmetry for Reflection Data Support
              O1
```

Author Response: its very unlikely that we have discoverd a new polymorph of ice

Alert level C

```
ABSMU01_ALERT_1_C  The ratio of given/expected absorption coefficient lies
               outside the range 0.99 <> 1.01
            Calculated value of mu =     0.101
            Value of mu given      =     0.102
PLAT042_ALERT_1_C Calc. and Reported MoietyFormula Strings  Differ     Please Check
              Calc: H2 O
              Rep.: H2 O1
PLAT053_ALERT_1_C Minimum Crystal Dimension Missing (or Error) ...     Please Check
PLAT054_ALERT_1_C Medium  Crystal Dimension Missing (or Error) ...     Please Check
PLAT055_ALERT_1_C Maximum Crystal Dimension Missing (or Error) ...     Please Check
PLAT923_ALERT_1_C S    Values in the CIF and FCF Differ by ......     -0.011 Check
```

Alert level G

```
PLAT110_ALERT_2_G ADDSYM Detects Potential Lattice Translation ...          ? Check
PLAT112_ALERT_2_G ADDSYM Detects New (Pseudo) Symm. Elem        I        100 %Fit
PLAT116_ALERT_2_G ADDSYM Included (Pseudo) Lattice Translation ...     Please Check
PLAT152_ALERT_1_G The Supplied and Calc. Volume s.u. Differ by ...          6 Units
PLAT300_ALERT_4_G Atom Site Occupancy of H1         Constrained at     0.4999 Check
PLAT304_ALERT_4_G Non-Integer Number of Atoms in ..... (Resd     1)      2.37 Check
PLAT883_ALERT_1_G Absent Datum for _atom_sites_solution_primary ..     Please Do !
PLAT910_ALERT_3_G Missing # of FCF Reflection(s) Below Theta(Min).          1 Note
```

```
                0  1  1,
PLAT912_ALERT_4_G Missing # of FCF Reflections Above STh/L=  0.600          7 Note
PLAT913_ALERT_3_G Missing # of Very Strong Reflections in FCF ....          1 Note
                0  1  1,
PLAT933_ALERT_2_G Number of HKL-OMIT Records in Embedded .res File          1 Note
                0  1  1,
PLAT958_ALERT_1_G Calculated (ThMax) and Actual (FCF) Lmax Differ.          3 Units
PLAT960_ALERT_3_G Number of Intensities with I < - 2*Sigma(I) ....          1 Check
PLAT969_ALERT_5_G The 'Henn et al.' R-Factor-gap value ..........       2.494 Note
              Predicted wR2: Based on SigI**2  1.69 or SHELX Weight  2.94
PLAT979_ALERT_1_G NoSpherA2 Scattering Factors Used .............     Please Note
```

```
  1 ALERT level A = Most likely a serious problem - resolve or explain
  2 ALERT level B = A potentially serious problem, consider carefully
  6 ALERT level C = Check. Ensure it is not caused by an omission or oversight
 15 ALERT level G = General information/check it is not something unexpected

 10 ALERT type 1 CIF construction/syntax error, inconsistent or missing data
  5 ALERT type 2 Indicator that the structure model may be wrong or deficient
  5 ALERT type 3 Indicator that the structure quality may be low
  3 ALERT type 4 Improvement, methodology, query or suggestion
  1 ALERT type 5 Informative message, check
```

It is advisable to attempt to resolve as many as possible of the alerts in all categories. Often the minor alerts point to easily fixed oversights, errors and omissions in your CIF or refinement strategy, so attention to these fine details can be worthwhile. In order to resolve some of the more serious problems it may be necessary to carry out additional measurements or structure refinements. However, the purpose of your study may justify the reported deviations and the more serious of these should normally be commented upon in the discussion or experimental section of a paper or in the "special_details" fields of the CIF. checkCIF was carefully designed to identify outliers and unusual parameters, but every test has its limitations and alerts that are not important in a particular case may appear. Conversely, the absence of alerts does not guarantee there are no aspects of the results needing attention. It is up to the individual to critically assess their own results and, if necessary, seek expert advice.

Publication of your CIF in IUCr journals

A basic structural check has been run on your CIF. These basic checks will be run on all CIFs submitted for publication in IUCr journals (*Acta Crystallographica*, *Journal of Applied Crystallography*, *Journal of Synchrotron Radiation*); however, if you intend to submit to *Acta Crystallographica Section C* or *E* or *IUCrData*, you should make sure that full publication checks are run on the final version of your CIF prior to submission.

Publication of your CIF in other journals

Please refer to the *Notes for Authors* of the relevant journal for any special instructions relating to CIF submission.

PLATON version of 11/11/2024; check.def file version of 11/11/2024

Datablock solve2 - ellipsoid plot

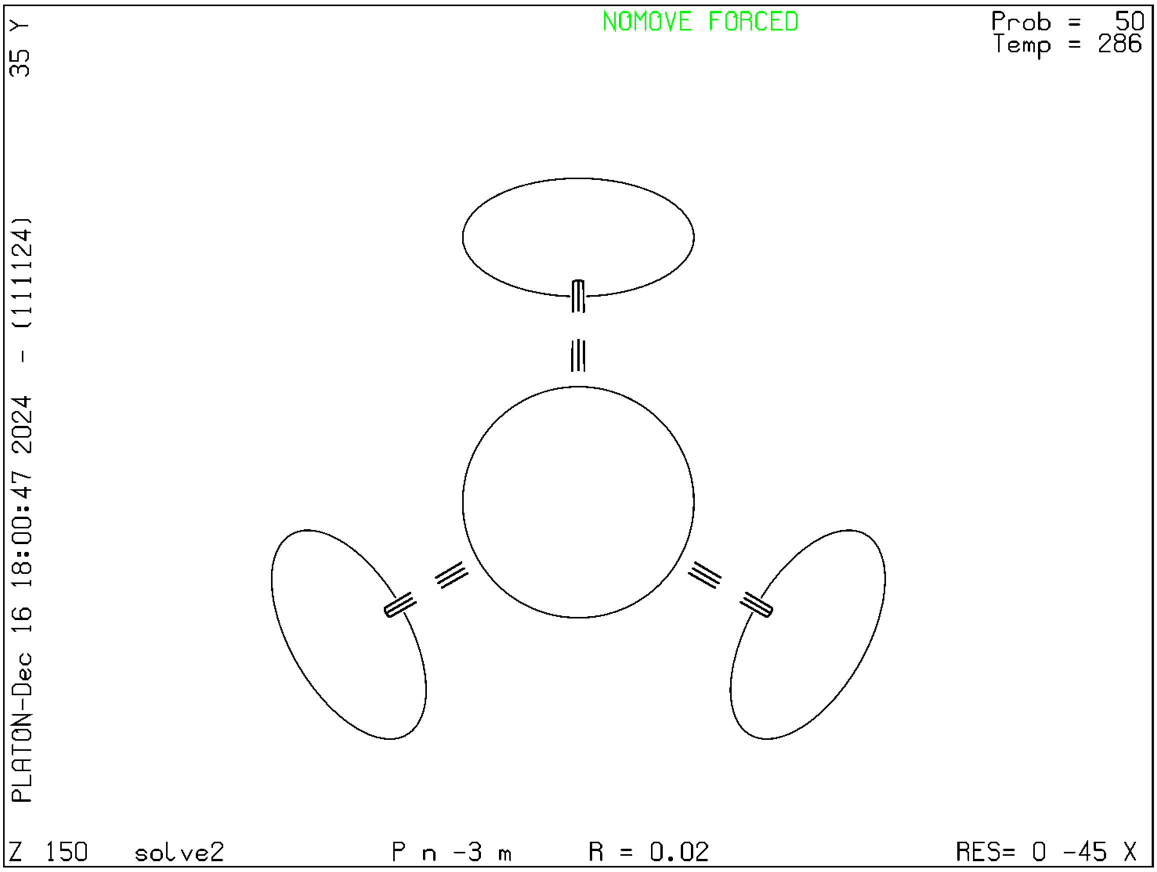

Supplement: Supplementary file 1 [file d2o_single_site_checkcif.pdf › cify/d2o/d2o_single_site_checkcif.pdf]
