# Supplementary material for: Structure of ice VII with Hirshfeld atom refinement
Source: IUCrJ. 2025 Apr 25;12(Pt 3):288–94. doi: 10.1107/S2052252525002581 (PMC12044852; doi:10.1107/S2052252525002581)

checkCIF/PLATON report

Structure factors have been supplied for datablock(s) iceVIID2O_0m

THIS REPORT IS FOR GUIDANCE ONLY. IF USED AS PART OF A REVIEW PROCEDURE FOR PUBLICATION, IT SHOULD NOT REPLACE THE EXPERTISE OF AN EXPERIENCED CRYSTALLOGRAPHIC REFEREE.

No syntax errors found. CIF dictionary Interpreting this report

Datablock: iceVIID2O_0m

```
Bond precision:   = 0.0000 A                Wavelength=0.43400

Cell:                a=3.3661(4)      b=3.3661(4)      c=3.3661(4)
                     alpha=90         beta=90          gamma=90
Temperature:         273 K

                 Calculated                  Reported
Volume           38.140(14)                  38.140(8)
Space group      P n -3 m                    P n -3 m
Hall group       -P 4bc 2bc                  -P 4bc 2bc
Moiety formula   H2 O                        H2 O1
Sum formula      H2 O                        H2 O
Mr               18.02                       18.01
Dx,g cm-3        1.569                       1.569
Z                2                           2
Mu (mm-1)        0.065                       0.062
F000             20.0                        20.0
F000'            20.00
h,k,lmax         7,7,7                       3,7,4
Nref             50                          24
Tmin,Tmax        0.985,0.988
Tmin'            0.982

Correction method= Not given

Data completeness= 0.480           Theta(max)= 27.700

                                                    wR2(reflections)=
R(reflections)= 0.0597( 16)                         0.2069( 24)

S = 1.005                 Npar= 4
```

The following ALERTS were generated. Each ALERT has the format
test-name_ALERT_alert-type_alert-level.
Click on the hyperlinks for more details of the test.

Alert level A

ATOM007_ALERT_1_A _atom_site_aniso_label is missing
Unique label identifying the atom site.

Author Response: there are no atoms with anisotropic displacement paramters

PLAT029_ALERT_3_A _diffrn_measured_fraction_theta_full value Low . 0.636 Why?

Author Response: It is caused by the fact that sample was closed in Diamond Anvil Cell and as a result access to the reciprocal psace was restricted.

Alert level B

DIFMN02_ALERT_2_B The minimum difference density is < -0.1*ZMAX*1.00
_refine_diff_density_min given = -1.159
Test value = -0.800

Author Response: quality of this dataset is relatively poor

PLAT088_ALERT_3_B Poor Data / Parameter Ratio .... 6.00 Note

Author Response: low number of reflections has been measured

PLAT098_ALERT_2_B Large Reported Min. (Negative) Residual Density -1.16 eA-3

Author Response: quality of this dataset is relatively poor

PLAT113_ALERT_2_B ADDSYM Suggests Possible Pseudo/New Space Group Im-3m Check
Check Model Parameter Symmetry for Reflection Data Support
O1
PLAT911_ALERT_3_B Missing FCF Refl Between Thmin & STh/L= 0.600 4 Report
1 1 1, 0 2 2, 1 2 2, 1 1 3,

Author Response: It is caused by the fact that sample was closed in Diamond Anvil Cell and as a result access to the reciprocal psace was restricted.

Alert level C

DIFMN03_ALERT_1_C The minimum difference density is < -0.1*ZMAX*0.75
The relevant atom site should be identified.

```
PLAT042_ALERT_1_C Calc. and Reported MoietyFormula Strings  Differ     Please Check
              Calc: H2 O
              Rep.: H2 O1
PLAT905_ALERT_3_C Negative K value in the Analysis of Variance ...     -0.006 Report
PLAT923_ALERT_1_C S    Values in the CIF and FCF Differ by .......     -0.025 Check
```

Alert level G

```
ABSMU01_ALERT_1_G  Calculation of _exptl_absorpt_correction_mu
                not performed for this radiation type.
PLAT110_ALERT_2_G ADDSYM Detects Potential Lattice Translation ...          ? Check
PLAT112_ALERT_2_G ADDSYM Detects New (Pseudo) Symm. Elem         I        100 %Fit
PLAT116_ALERT_2_G ADDSYM Included (Pseudo) Lattice Translation ...     Please Check
PLAT152_ALERT_1_G The Supplied and Calc. Volume s.u. Differ by ...          6 Units
PLAT199_ALERT_1_G Reported _cell_measurement_temperature ..... (K)        273 Check
PLAT200_ALERT_1_G Reported   _diffrn_ambient_temperature ..... (K)        273 Check
PLAT300_ALERT_4_G Atom Site Occupancy of H          Constrained at     0.4999 Check
PLAT304_ALERT_4_G Non-Integer Number of Atoms in ..... (Resd    1)       2.37 Check
PLAT883_ALERT_1_G Absent Datum for _atom_sites_solution_primary ..     Please Do !
PLAT912_ALERT_4_G Missing # of FCF Reflections Above STh/L=  0.600         23 Note
PLAT958_ALERT_1_G Calculated (ThMax) and Actual (FCF) Lmax Differ.          3 Units
PLAT969_ALERT_5_G The 'Henn et al.' R-Factor-gap value ..........      7.731 Note
              Predicted wR2: Based on SigI**2  2.68 or SHELX Weight 20.10
PLAT979_ALERT_1_G NoSpherA2 Scattering Factors Used .............     Please Note
```

```
   2 ALERT level A = Most likely a serious problem - resolve or explain
   5 ALERT level B = A potentially serious problem, consider carefully
   4 ALERT level C = Check. Ensure it is not caused by an omission or oversight
  14 ALERT level G = General information/check it is not something unexpected

  11 ALERT type 1 CIF construction/syntax error, inconsistent or missing data
   6 ALERT type 2 Indicator that the structure model may be wrong or deficient
   4 ALERT type 3 Indicator that the structure quality may be low
   3 ALERT type 4 Improvement, methodology, query or suggestion
   1 ALERT type 5 Informative message, check
```

It is advisable to attempt to resolve as many as possible of the alerts in all categories. Often the minor alerts point to easily fixed oversights, errors and omissions in your CIF or refinement strategy, so attention to these fine details can be worthwhile. In order to resolve some of the more serious problems it may be necessary to carry out additional measurements or structure refinements. However, the purpose of your study may justify the reported deviations and the more serious of these should normally be commented upon in the discussion or experimental section of a paper or in the "special_details" fields of the CIF. checkCIF was carefully designed to identify outliers and unusual parameters, but every test has its limitations and alerts that are not important in a particular case may appear. Conversely, the absence of alerts does not guarantee there are no aspects of the results needing attention. It is up to the individual to critically assess their own results and, if necessary, seek expert advice.

Publication of your CIF in IUCr journals

A basic structural check has been run on your CIF. These basic checks will be run on all CIFs submitted for publication in IUCr journals (*Acta Crystallographica*, *Journal of Applied Crystallography*, *Journal of Synchrotron Radiation*); however, if you intend to submit to *Acta Crystallographica Section C* or *E* or *IUCrData*, you should make sure that full publication checks are run on the final version of your CIF prior to submission.

Publication of your CIF in other journals

Please refer to the *Notes for Authors* of the relevant journal for any special instructions relating to CIF submission.

Validation response form

Please find below a validation response form (VRF) that can be filled in and pasted into your CIF.

```
# start Validation Reply Form
_vrf_PLAT113_iceVIID2O_0m
;
PROBLEM: ADDSYM Suggests Possible Pseudo/New  Space Group      Im-3m Check
RESPONSE: ...
;
# end Validation Reply Form
```

PLATON version of 11/11/2024; check.def file version of 11/11/2024

Datablock iceVIID2O_0m - ellipsoid plot

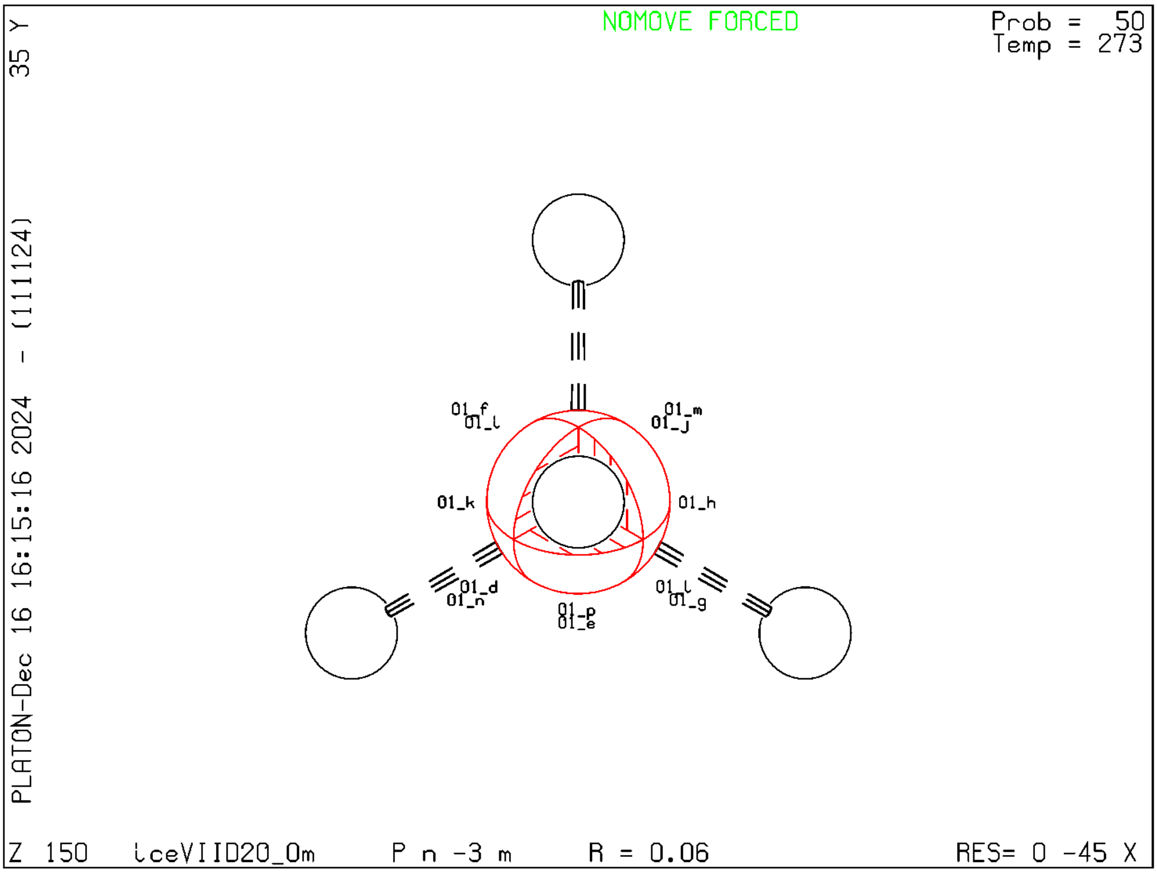

Supplement: Supplementary file 1 [file checkcif.pdf › cify/d2o_low_res/checkcif.pdf]
