# Supplementary material for: Structure of ice VII with Hirshfeld atom refinement
Source: IUCrJ. 2025 Apr 25;12(Pt 3):288–94. doi: 10.1107/S2052252525002581 (PMC12044852; doi:10.1107/S2052252525002581)

checkCIF/PLATON report

Structure factors have been supplied for datablock(s) IceVII_DAC2_0m

THIS REPORT IS FOR GUIDANCE ONLY. IF USED AS PART OF A REVIEW PROCEDURE FOR PUBLICATION, IT SHOULD NOT REPLACE THE EXPERTISE OF AN EXPERIENCED CRYSTALLOGRAPHIC REFEREE.

No syntax errors found. CIF dictionary Interpreting this report

Datablock: IceVII_DAC2_0m

Bond precision: = 0.0000 A Wavelength=0.43400

Cell:	a=3.3891(3)	b=3.3891(3)	c=3.3891(3)
	alpha=90	beta=90	gamma=90
Temperature:	273 K		

	Calculated	Reported
Volume	38.927(10)	38.927(6)
Space group	P n -3 m	P n -3 m
Hall group	-P 4bc 2bc	-P 4bc 2bc
Moiety formula	0.007(H12 O6)	2(H1 O0.5)
Sum formula	H0.08 O0.04	H2 O
Mr	0.75	18.01
Dx,g cm-3	1.537	1.537
Z	48	2
Mu (mm-1)	0.064	0.061
F000	20.0	20.0
F000'	20.01	
h,k,lmax	8,8,8	2,8,5
Nref	66	45
Tmin,Tmax		
Tmin'		

Correction method= Not given

Data completeness= 0.682 Theta(max)= 31.340

R(reflections)= 0.0234(28) wR2(reflections)= 0.0824(45)

S = 1.977 Npar= 6

The following ALERTS were generated. Each ALERT has the format
test-name_ALERT_alert-type_alert-level.
Click on the hyperlinks for more details of the test.

Alert level A

```
ATOM007_ALERT_1_A  _atom_site_aniso_label is missing
            Unique label identifying the atom site.
```

Author Response: only isotropic displacement paramters are used

```
PLAT029_ALERT_3_A _diffrn_measured_fraction_theta_full value Low .      0.818 Why?
```

Author Response: It is caused by the fact that sample was closed in Diamond Anvil Cell and as a result access to the reciprocal psace was restricted.

Alert level B

```
PLAT088_ALERT_3_B Poor Data / Parameter Ratio ...................       7.50 Note
```

Author Response: low number of reflections has been measured, pourpose of the refinement was to check results of the refinement for the model used inspite of the poor Data / Parameter Ratio

---

Alert level C

```
PLAT041_ALERT_1_C Calc. and Reported SumFormula    Strings  Differ     Please Check
              Calc: H0.08 O0.04
              Rep.: H2 O
PLAT042_ALERT_1_C Calc. and Reported MoietyFormula Strings  Differ     Please Check
              Calc: 0.007(H12 O6)
              Rep.: 2(H1 O0.5)
PLAT053_ALERT_1_C Minimum Crystal Dimension Missing (or Error) ...     Please Check
PLAT054_ALERT_1_C Medium  Crystal Dimension Missing (or Error) ...     Please Check
PLAT055_ALERT_1_C Maximum Crystal Dimension Missing (or Error) ...     Please Check
PLAT921_ALERT_1_C R1  in the CIF and FCF Differ by ..............     0.0036 Check
PLAT923_ALERT_1_C S    Values in the CIF and FCF Differ by .......     -0.053 Check
```

Alert level G

```
ABSMU01_ALERT_1_G  Calculation of _exptl_absorpt_correction_mu
                not performed for this radiation type.
PLAT045_ALERT_1_G Calculated and Reported Z Differ by a Factor ...        24 Check
PLAT152_ALERT_1_G The Supplied and Calc. Volume s.u. Differ by ...         4 Units
PLAT199_ALERT_1_G Reported _cell_measurement_temperature ..... (K)       273 Check
PLAT200_ALERT_1_G Reported   _diffrn_ambient_temperature ..... (K)       273 Check
PLAT300_ALERT_4_G Atom Site Occupancy of O1         Constrained at    0.1667 Check
PLAT300_ALERT_4_G Atom Site Occupancy of H1         Constrained at    0.1667 Check
PLAT301_ALERT_3_G Main Residue  Disorder .............(Resd    1)       100% Note
```

```
PLAT883_ALERT_1_G Absent Datum for _atom_sites_solution_primary ..     Please Do !
PLAT910_ALERT_3_G Missing # of FCF Reflection(s) Below Theta(Min).          1 Note
                0  1  1,
PLAT912_ALERT_4_G Missing # of FCF Reflections Above STh/L=  0.600         20 Note
PLAT913_ALERT_3_G Missing # of Very Strong Reflections in FCF ....          1 Note
                0  1  1,
PLAT933_ALERT_2_G Number of HKL-OMIT Records in Embedded .res File          1 Note
                0  6  2,
PLAT958_ALERT_1_G Calculated (ThMax) and Actual (FCF) Lmax Differ.          3 Units
PLAT969_ALERT_5_G The 'Henn et al.' R-Factor-gap value ..........       2.345 Note
              Predicted wR2: Based on SigI**2  3.51 or SHELX Weight  4.06
PLAT979_ALERT_1_G NoSpherA2 Scattering Factors Used .............     Please Note
```

```
   2 ALERT level A = Most likely a serious problem - resolve or explain
   1 ALERT level B = A potentially serious problem, consider carefully
   7 ALERT level C = Check. Ensure it is not caused by an omission or oversight
  16 ALERT level G = General information/check it is not something unexpected

  16 ALERT type 1 CIF construction/syntax error, inconsistent or missing data
   1 ALERT type 2 Indicator that the structure model may be wrong or deficient
   5 ALERT type 3 Indicator that the structure quality may be low
   3 ALERT type 4 Improvement, methodology, query or suggestion
   1 ALERT type 5 Informative message, check
```

It is advisable to attempt to resolve as many as possible of the alerts in all categories. Often the minor alerts point to easily fixed oversights, errors and omissions in your CIF or refinement strategy, so attention to these fine details can be worthwhile. In order to resolve some of the more serious problems it may be necessary to carry out additional measurements or structure refinements. However, the purpose of your study may justify the reported deviations and the more serious of these should normally be commented upon in the discussion or experimental section of a paper or in the "special_details" fields of the CIF. checkCIF was carefully designed to identify outliers and unusual parameters, but every test has its limitations and alerts that are not important in a particular case may appear. Conversely, the absence of alerts does not guarantee there are no aspects of the results needing attention. It is up to the individual to critically assess their own results and, if necessary, seek expert advice.

Publication of your CIF in IUCr journals

A basic structural check has been run on your CIF. These basic checks will be run on all CIFs submitted for publication in IUCr journals (*Acta Crystallographica*, *Journal of Applied Crystallography*, *Journal of Synchrotron Radiation*); however, if you intend to submit to *Acta Crystallographica Section C* or *E* or *IUCrData*, you should make sure that full publication checks are run on the final version of your CIF prior to submission.

Publication of your CIF in other journals

Please refer to the *Notes for Authors* of the relevant journal for any special instructions relating to CIF submission.

PLATON version of 11/11/2024; check.def file version of 11/11/2024

Datablock IceVII_DAC2_0m - ellipsoid plot

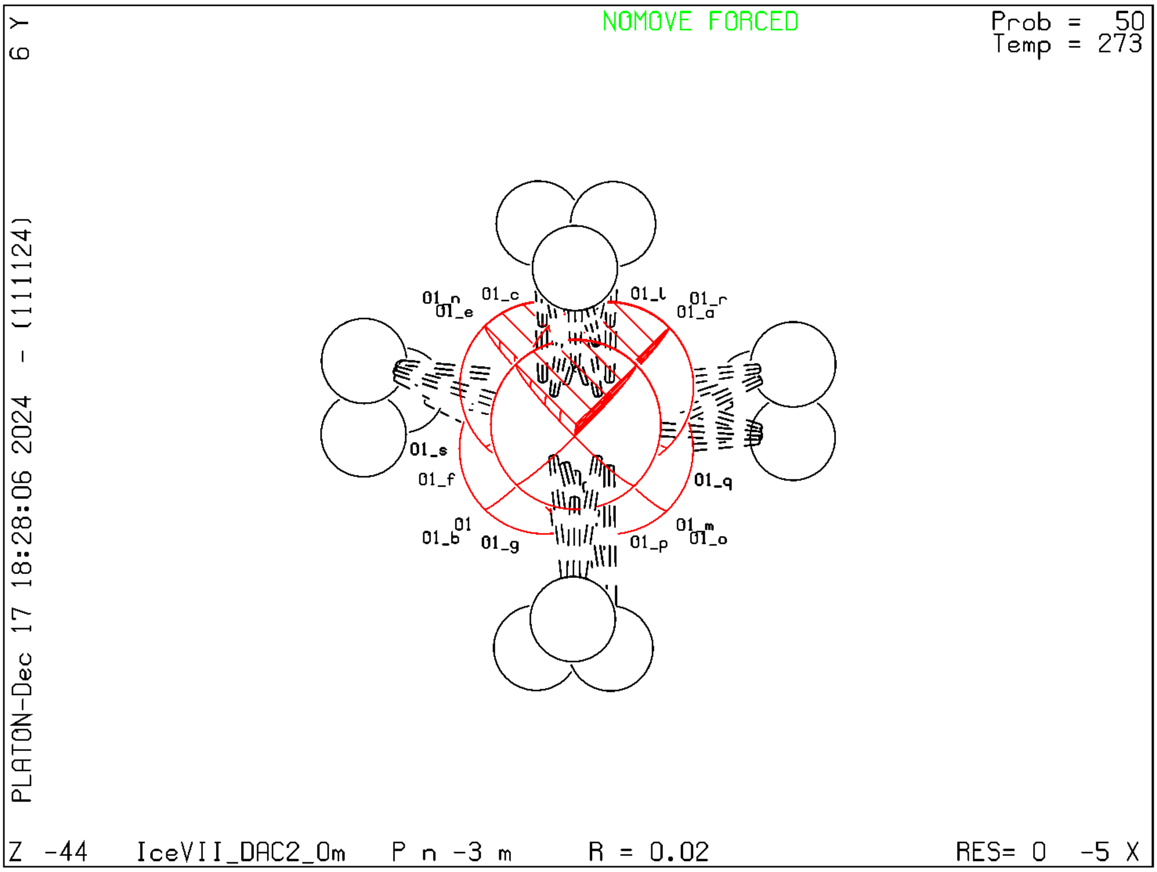

Supplement: Supplementary file 1 [file mix_O_100_H_z_gt_x_checkcif.pdf › cify/mix/mix_O_100_H_z_gt_x_checkcif.pdf]
